# *Clostridium autoethanogenum* protein inclusion in the diet for broiler: Enhancement of growth performance, lipid metabolism, and gut microbiota

**DOI:** 10.3389/fvets.2022.1028792

**Published:** 2022-11-24

**Authors:** Yushan Wu, Jing Wang, Ming Jia, Shixin Huang, Ying Cao, Ting Yao, Junguo Li, Yuxin Yang, Xu Gu

**Affiliations:** ^1^Institute of Feed Research of Chinese Academy of Agricultural Sciences, Laboratory of Feed-Derived Factor Risk Assessment for Animal Product Quality and Safety, Ministry of Agriculture and Rural Affairs, Beijing, China; ^2^Shanghai Municipal Supervisory Institute Veterinary Drugs and Feedstaff, Shanghai, China; ^3^College of Animal Science and Technology, Northwest A&F University, Xianyang, China

**Keywords:** cobb broiler, *Clostridium autoethanogenum* protein, biochemical indexes, lipid metabolism, gut microbiota

## Abstract

This study aimed to investigate the effects of dietary supplementation of the new single-cell protein *Clostridium autoethanogenum* protein (CAP) on growth performance, plasma biochemical indexes, liver histology, lipid metabolism, and gut microbiota in Cobb broilers. According to the randomized block experimental design, 960 Cobb broilers (1d old) were divided into six treatments with eight replicates of 20 birds each. Six isonitrogenous and isoenergetic diets were formulated with different contents of CAP (0, 1, 2, 3, 4, and 5%) to replace soybean meal (SBM). The results showed that the addition of CAP did not influence liver health when it exceeded 2%. The protein metabolism markers and feed conversion rate increased (*P* < 0.05), significantly improving the growth performance. When the content of CAP was greater than 4%, it could promote lipolysis without affecting lipogenesis, decreasing the abdominal fat rate. There was no significant difference in MDA between these groups (*P* = 0.948). The increase of SOD and GSH-Px indicated the enhancement of antioxidant response. Alpha diversity did not significantly differ between groups (*P* > 0.05). Inclusion of 4% or less CAP led to the increase in beneficial microbiota, the concentration of short-chain fatty acids (SCFAs) such as acetic acid, propionic acid, and butyric acid (*P* < 0.05), and the concentration of primary bile acids such as cholic acid and goose deoxycholic acid (*P* < 0.05). While the concentration of secondary bile acids such as taurocholic acid and taurine goose deoxycholic acid was decreased (*P* < 0.05). These results illustrated that the CAP had a high potential for application in poultry nutrition. In terms of improving growth performance and antioxidant capacity and reducing fat deposition rate, 4% CAP content is recommended.

## Introduction

As the most commonly used plant-based protein in the feed industry, SBM is widely used with the advantages of fewer anti-nutritional factors, amino acid balance, and various vitamins in poultry farming (1–3). However, in recent years, because of the high price and the source of SBM mainly on import, the protein feed resources in China have become a disgrace with the rapid development of the poultry industry. This trend suggests that traditional protein can no longer alleviate the pressure of protein shortage these days (4). Therefore, while improving the utilization of existing protein resources, searching for new cost-effective protein resources to gradually reduce and replace the expensive feed resources like SBM has become an important issue in poultry farming and an effective way to alleviate the shortage of feeding protein resources.

Single-cell protein (SCP) is also called microbial or bacterial protein. The concept of SCP was first proposed at MIT in the 20th century, which used to represent microbial protein for food and feed production by a large-scale culture of yeast or bacteria. SCP can be obtained by culturing bacteria, yeasts, molds, algae, and basidiomycetes with various substrates (5–9) and has various benefits such as short culture cycle, high crude protein, and rich vitamins (10, 11). Meanwhile, it can increase palatability efficiency, opening up numerous opportunities for production and use as a viable alternative to animal-based protein sources. Much attention has been paid to the application of SCP in animal feed. In the research about SCP on poultry breeding, several studies have been reported, which focus on chlorella (12), date palm fermentation protein (13), yeast single cell protein (14), and autolyzed saccharomyces cerevisiae (15). All of them positively affect the health of Cobb broilers, but it is necessary to pay attention to the proportion of SCP when using it. Otherwise, serious death of broilers may be caused (16). Due to the particularity of the poultry digestive system (17), the study of SCP for poultry farming is of great importance (18).

*Clostridium autoethanogenum* protein (CAP) is a new type of bacterial protein. It can be produced industrially with carbon monoxide as a carbon source and under the fermentation of *Clostridium autoethanogenum* (CA). Because the carbon source can be derived from industrial waste gas, using industrial waste effectively alleviates the environmental problems caused by harmful gas emissions and contributes to achieving carbon neutrality. As one of the main by-products in ethanol fermentation, it is green, low-carbon, energy-saving, and environmentally friendly. The “out of nothing” feed protein is critically important, and CAP's abundance of organic proteins helps realize this transformation of by-products into something of value. In addition, for every 10,000 tons of ethanol produced, about 1,500 tons of bacterial protein could be obtained (19), proving that it has the characteristics of high yield and improving quality and efficiency. CAP has a crude protein level of 83.2%, is rich in trace minerals and vitamins, and lacks anti-nutritional components. The essential amino acid balance is similar to that of fish meal (FM) and higher than that of SBM (19, 20).

Furthermore, CAP is rich in methionine, the first limiting amino acid required by chickens, which can make up for the deficiency of methionine in SBM (21). Taken together, the CAP can be used as a potential protein source for animals. CAP has been widely studied in aquaculture animals. The potential value of CAP in several species has been assessed, including grass carp (19), large-mouth bass (22, 23), Pacific white shrimp (24), Tilapia juveniles (25, 26), and juvenile Jian carp (27). An appropriate proportion of SBM without adverse effects on the growth of the above aquaculture animals could enhance their growth performance.

Despite its potential as a feed protein, CAP has yet to be studied in the context of a broiler diet, and its use in actual production remains limited. With that in mind, this study used a gradient addition experiment in which CAP was substituted for SBM to learn more about how CAP affects the health and growth of Cobb broilers and their biochemistry, hepatic histopathology, lipid metabolism, and gut microbiota.

## Materials and methods

### Materials

During the feeding period, the experimental broilers were maintained in compliance with the Laboratory Animal Welfare Guidelines of China (Decree No. 2 of the Ministry of Science and Technology, issued in 1988). All experimental procedures were performed in accordance with (the Cobb broiler feeding management manual) and the Regulations for the Administration of Affairs Concerning Experimental Animals. Animal experiments were conducted at the Nankou Base of the Chinese Academy of Agricultural Sciences. CAP used in the experiment is a light yellow powder provided by Beijing Shoulang Biotechnology Co., Ltd. The crude protein content is 84.69%, and the digestibility of pepsin is 90.2%.

### Experimental design and diets

At the beginning of the experiment, a total of 960 one-day-old broilers (male of Cobb) with an average initial weight of 41.43nt (male of the Co-treatment group had eight replicates pens with 20 chickens per pen). The experiment lasted for 6 weeks and was divided into two stages, feeding the early feed for 1 to 21 d and the late feed for 22 to 42 d. The ingredients and calculated chemical composition of the basal diet are shown in Supplementary Table S1.

The basic diet was given to the control group (T0), and the experimental groups (T1, T2, T3, T4, and T5) were given 1, 2, 3, 4, and 5% CAP, respectively, to replace 6, 12, 18, 25, and 31% of SBM in the basic beginning diet and 8, 15, 22, 29, and 36% of SBM, respectively. The basal diet met the NRC (28) recommended nutrient requirements for broiler chickens. Six experimental diets were kept to be isonitrogenous and isoenergetic. The amino acid composition of experimental diets is shown in Supplementary Table S1. Pelleted feed was prepared under the conditions of a ring mold aperture of 3.0 mm, and a length diameter ratio of 10:1.

All the chickens were reared in the same experimental room, housed in a room with the same automatic control artificial light (16:8, L/D) and light intensity (20 lx). The relative humidity of the experimental house was maintained at 60%. The room temperature remained at 33 chickens reared in the same experimental room. For the first three days, the temperature in the room stayed at 33°C, and on the fourth day, it was lowered to 25°C. Chickens were vaccinated in accordance with the immunization program. All diets were in granular form. During the experiment, feed and water were fed at will.

### Composition analysis

Dietary crude protein, crude lipid, moisture, and ash were determined using AOAC standard methods (2006). Dry matter was analyzed by drying the samples to constant weight at 105°C. Crude protein was determined with a Kjeltec TM 2300 Unit (FOSS, Denmark) using the Kjeldahl method, and crude protein content was estimated by multiplying nitrogen by 6.25. Crude lipid was analyzed using a Soxtec System HT 1047 Hydrolyzing Unit (FOSS, Denmark), followed by Soxhlet extraction using a Soxtec System 1043 (FOSS, Denmark). Ash was analyzed by combustion in a CF 1100 muffle furnace (Carbolite, UK) at 550°C for 6 h. All actual measured values are listed in Supplementary Table S2.

### Growth performance and slaughter performance

Two nutritional phases, including the beginning phase (1 to 21 d) and the final phase (22 to 42 d), were designed for this experiment (Supplementary Table S2). At the end of 3 and 6 weeks of age, chickens (after a 12-h fast) were weighed, and feed intake was recorded for each treatment pen for the calculation of average final body weight (FBW), body weight gain (BWG), average daily feed intake (ADFI), and feed-to-gain ratio (F/G). Mortality and culling were recorded daily for each pen. Dead chickens were weighed, and the weights were used to correct the F/G of chickens.

At the 21 and 42 d, blood samples were collected from 48 broilers (one bird per cage and eight birds per treatment) *via* bleeding from the jugular vein. The broilers to be slaughtered were weighed separately and killed by cervical dislocation. After obtaining the blood sample, it was centrifuged at 3000 g at 4°C for 15 min (Becton Dickinson Vacutainer Systems, Franklin Lakes, NJ). In this way, the plasma is obtained. Then plasma was stored below −20°C. The leg, breast, and abdominal fat were harvested and weighed to calculate the relative organ weights (% of live BW).

### Assay of serum metabolites

Triglyceride (TG), total cholesterol (TC), total protein (TP), albumin (ALB), uric acid (UA) activities and the enzymatic activities of Superoxide Dismutase (SOD), Glutathione Peroxidase (GSH-Px), Catalase (CAT) and the Malondialdehyde (MDA) were determined by assay kits (Nanjing jiancheng Co., Nanjing, China). The numbers of the kit are shown in Supplementary Table S3.

### RNA isolation, reverse transcription, and mRNA levels analysis

Total RNA was isolated from the liver using RNAiso Plus reagent (Takara, Japan), spectrophotometrically quantified using a NanoDrop 2000 (Thermo, USA), and electrophoresed on a 1% denaturing agarose gel to test the integrity. Refer to the instructions for the specific operation process. The core fragments of all the genes were obtained from the database of RNA-Seq. β-actin (GenBank accession no. NM2055181), a housekeeping gene whose expression was found to be unaffected by the treatment in the present experiment, was used as an endogenous reference to normalize the template amount. The gene-specific primers used for mRNA quantification by RT-qPCR are shown in Table 1. Detection process and setting reference Wei et al. (19).

**Table 1:** Primer sequences for real-time PCR.

**Gene**	**Forward primer (5^′^-3^′^)**	**Reverse primer (5^′^-3^′^)**	**Product size (bp)**	**E-Values (%)**	**TM (°C)**
β-actin	TGTTACCAACACCCACACCC	AGACTGCTGCTGACACCTTC	148	99.5	58.5
FASN	TGGTTGACTGCCACCAATTG	ACCCCACTTCCATCACGAT	213	93.0	57.2
ACC1	GCTGGGTTGAGCGACTAATG	GGGAAACTGGCAAAGGACTG	173	98.5	57.2
CPT-1	GACGGACACTGCAAAGGAGA	GGCATCAGGGCTGGTTTTTG	204	97.7	57.5
ATGL	TCACCTTCAGCGTCCAAGTC	GCACATGCCTCCAAAAGAGC	186	92.8	57.9
PPARα	ACGAATGCCAAGGTCTGAGA	TGCAAGGATGACTCTGGCTT	169	96.2	57.5

FASN, fatty acid synthase; ACC 1, acetyl-CoA carboxylase 1; CPT-1, Carnitine Palmitoyltransferase; ATGL, adipose triglyceride lipase; and PPAR, peroxisome proliferator-activated receptor.

### Histopathological examination of the liver

First, the liver samples were fixed in 4% paraformaldehyde for 24 h. Subsequently, all liver samples were dehydrated according to standard procedures. Then, the samples were embedded in paraffin. Cut to 6 μm after cooling and demolding section. Finally, the staining of liver sections was carried out according to the hema-toxylinandeosin (H & E) protocol. The stained sections were observed with an optical LAS V4.0 light microscope (Leica DM2500, Leica, Germany). After confirming successful staining, we applied the data.

### Composition and association analysis of gut microbiota

Genomic DNA of the valve intestines was extracted using a PowerSoil DNA1 Isolation Kit (MoBio Laboratories, Carlsbad, CA) following the manual. The purity and quality of the genomic DNA were checked on 0.8% agarose gels. The V3-V4 hypervariable region of the seven bacterial 16S rRNA gene was amplified with the primers 338F (ACTCCTACGGGAGGCAGCAG) and 806R (GGACTACHVGGGTWTCTAAT). The PCR products were purified using a QIAquick Gel Extraction Kit (QIAGEN, Germany), quantified using real-time PCR, and performed deep sequencing on a MiSeq platform (San Diego, USA). Quality control of the raw data was performed by FastQC (version 0.19.6). Qualified reads were separated using the sample-specific barcode sequences and denoised with DADA2, then the sequences were clustered into the ASVs at a similarity level of 100%. Based on the silva138/16s_bacteria RNA gene database, 16S rRNA target genes were sequenced, and taxonomy assignments were derived. QIIME2, R packages (version 3.3.1), and python (version 2.7) were mainly used to analyze the raw sequence dataset. All the raw 16S rRNA gene amplicon sequencing data are deposited in the NCBI SRA database under the BioProject accession number PRJNA877374.

### SCFAs quantitative analysis

SCFAs in plasma and cecum samples were determined as the description by Feng et al. (29). SCFAs were quantify with UHPLC-QqQ-MS/MS after 5-(dimethylamino)-1-carbohydrazide-isoquinoline (DMAQ) derivatization. Chromatographic separation was achieved on a UPLC BEH C18 column (2.1 × 150 mm, 1.7 μm particle size, pore size: 130 Å). A binary gradient was used with mobile phase A of deionized water and mobile phase B of ACN. Both mobile phases contained 1% formic acid. MS acquisition was performed in the positive ESI mode.

### Bile acids quantitative analysis

Plasma and bile samples were prepared in accordance with a previous report (30). The eluted substances of ultra-performance liquid chromatography systems coupled with a triple-quadrupole mass spectrometer (UHPLC-QqQ-MS/MS) were ionized in an electrospray ionization source, the negative mode (ESI-). Chromatographic separation was operated on a UPLC BEH C18 column (100 × 2.1 mm, 1.7 μm). The mobile phase consisted of water in 0.1% formic acid (A) and acetonitrile in 0.1% formic acid (B). The gradient elution was applied, and MS detection was conducted in the negative mode. Standards for all BAs were used to identify the different BA metabolites detected by UHPLC-MS/MS. The Agilent Mass Hunter software (version B.08.00) was used to control instruments and acquire data. The raw data were processed by Agilent Mass Hunter Workstation Software (version B.08.00) using the default parameters and assisting manual inspection in ensuring each compound's qualitative and quantitative accuracies. The peak areas of target compounds were integrated and output for quantitative calculations.

### Statistical analyses

The normal distribution of all data was tested before statistical Analysis. The mean and significant differences between groups were performed using the social science statistical software package (SPSS, version 20.0). We used a one-way analysis of variance (one-way ANOVA) and a non-parametric test (Kruskal–Wallis test) for comparing the data. *P* < 0.05 were considered to be statistically significant, and data were reported as mean value Kruskal–Wallis test) for comparison, tel (version 2010) was used for all graphics except those in the microbiological section, where R (version 3.3.1) was used.

## Results

### Growth performance and slaughter performance

Table 2 describes the effects of adding CAP on growth performance for the broiler. In the beginning, there were significant differences in the average daily feed intake (ADFI) and F/G (P 0.05) between the CAP addition groups, and throughout all the periods, the F/G was also considerably decreased (P 0.05). Compared with the control group, the supplementation of CAP had increased the body weight gain (BWG) in the beginning phase and overall phase of broilers. The T2 group increased significantly (*P* < 0.05). Other performance indicators had no significant differences (*P* > 0.05).

**Table 2:** Effects of adding CAP level on growth performance of White-feather Broilers (means ± SEM).

**Phase**	**Groups**	**T0**	**T1**	**T2**	**T3**	**T4**	**T5**	***P*-value**
The beginning	ADFI, g^b^	53.9 ± 0.75^d^	59.8 ± 0.37^a^	57.8 ± 0.19^c^	58.7 ± 0.31^b^	57.6 ± 0.30^c^	57.1 ± 0.30^c^	0.021
(1–21d)	F/G^d^	1.40 ± 0.03^a^	1.38 ± 0.03^b^	1.36 ± 0.02^c^	1.35 ± 0.02^c^	1.33 ± 0.02^c^	1.35 ± 0.01^c^	0.012
	BWG, g^c^	884.3 ± 2.32	949.6 ± 11.5	938.3 ± 5.74	950.2 ± 10.4	950.7 ± 3.96	924.8 ± 6.03	0.252
The end	ADFI, g	136.3 ± 0.53	140.1 ± 1.16	140.3 ± 0.68	139.9 ± 1.30	139.5 ± 1.23	140.9 ± 1.12	0.944
(22–42d)	F/G	2.05 ± 0.03	2.03 ± 0.02	1.94 ± 0.01	2.01 ± 0.01	1.99 ± 0.01	1.94 ± 0.02	0.231
	BWG, g	1404 ± 14.4	1455 ± 22.0	1518 ± 12.8	1462 ± 11.1	1479 ± 16.5	1530 ± 21.4	0.544
Overall	ADFI, g	94.2 ± 0.35	98.0 ± 0.48	97.4 ± 0.49	97.0 ± 0.85	96.9 ± 0.65	97.6 ± 0.51	0.336
(1–42d)	F/G	1.79 ± 0.07^a^	1.80 ± 0.08^a^	1.71 ± 0.02^c^	1.75 ± 0.03^b^	1.72 ± 0.03^c^	1.70 ± 0.04^d^	0.039
	BWG, g	2288 ± 14.8	2405 ± 13.9	2456 ± 13.9	2412 ± 19.9	2429 ± 19.0	2454 ± 23.5	0.451

a Values (mean White-feather Broilers (means ± SEM).pase; PPAR, peroxisome proliferator differences (*P* < 0.05).

b ADFI (average daily feed intake, g) = feed intake/days.

c BWG (body weight gain, g) = (Wf -Wi). Wf is the final total wight, and Wi is the initial total weight.

d F/G (feed-to-gain ratio) = ADG/ADFI.

The slaughtered performances are presented in Table 3. Dressing percentage showed no significant differences among groups (*P* = 0.701), while eviscerated yields of treatments were increased compared with in T4 and T0 (*P* = 0.042), and the treatment groups increased compared with the control group. There is no significant change in breast muscle yield (*P* = 0.424) and thigh muscle yield (*P* = 0.320). Although the abdominal fat yield of all treatments was reduced, T4 was significantly lower than T0 and T1 (*P* < 0.001).

**Table 3:** Effects of adding CAP level (%) on slaughtered performances of 1–42d White-feather Broilers (means ± SEM).

**Item^1^**	**T0**	**T1**	**T2**	**T3**	**T4**	**T5**	***P*-value**
Dressing percentage	92.4 ± 0.22	92.1 ± 0.27	91.8 ± 0.32	92.0 ± 0.68	91.5 ± 0.32	91.8 ± 0.39	0.701
Eviscerated yield^2^	73.3 ± 0.60^a^	74.3 ± 0.67^ab^	76.0 ± 1.12^ab^	74.7 ± 0.64^ab^	76.8 ± 0.69^b^	73.9 ± 0.72^ab^	0.042
Breast muscle yield	25.3 ± 0.94	24.2 ± 0.43	23.5 ± 0.49	24.4 ± 1.02	25.7 ± 0.58	24.5 ± 0.60	0.424
Thigh muscle yield	22.0 ± 0.89	21.7 ± 0.28	22.2 ± 0.79	21.6 ± 0.70	21.0 ± 0.56	19.9 ± 0.78	0.320
Abdominal fat yield	1.76 ± 0.08^b^	1.53 ± 0.09^b^	1.54 ± 0.17^ab^	1.44 ± 0.11^ab^	1.24 ± 0.13^a^	1.30 ± 0.13^ab^	<0.001

1 Data are the mean of eight replicates (one bird with a replicate on 42 d, respectively).

2 Eviscerated yields were calculated as the percentage of the body weight after fasting; breast muscle and leg muscle yields were calculated as percentages of eviscerated weight; abdominal fat = abdominal fat weight/(eviscerated weight + abdominal fat weight) × 100%.

a−d The same as the above table.

### Serum metabolites

The effects of adding CAP difference level on serum metabolism parameters were presented in Table 4. At the beginning (1–21d), the plasma TG, TC, and UA were no different between all groups. CAP-supplemented diets do not significantly increase ALB (*P* = 0.148) and TP (*P* = 0.103). At the end (22–42d), CAP-supplemented diets decreased TP (*P* = 0.048) and UA (*P* = 0.019) significantly. Besides, CAP-supplemented diets significantly decrease TG (*P* = 0.048). The plasma TC and ALB were no different among the groups. UA in treatment groups increased compared to the control group, and T3 was significantly higher than in the T0 group. The effects of adding CAP difference level on antioxidant parameters were presented in Table 5. At the beginning (1–21d), the activity of glutathione peroxidase (GSH-Px) in the treatment groups was significantly higher than that in the control group (*P* = 0.03), especially T3 and T4. The activity of superoxide dismutase (SOD) in all treatment groups was higher than that in the control group, and T2 and T3 were significant (*P* < 0.001). Malondialdehyde (MDA) addition group was significantly higher than the control group (*P* = 0.023), the highest in T2. Treatment groups decreased GSH-Px/MDA (*P* < 0.001) and SOD/MDA (*P* < 0.001) significantly. At the end (22–42d), the level of GSH-Px in T2 and T5 was significantly lower than in the other groups (*P* = 0.001). The activity of SOD in all the treatment groups was higher than in the control group, especially T1 (*P* = 0.024). There was no significant difference in MDA between groups (*P* = 0.948). GSH/MDA was significantly declining in all phases. SOD/MDA significantly declined in the beginning phase and increased dramatically in the final phase.

**Table 4:** Effects of adding CAP difference level on serum metabolism parameters of white-feather broilers (means ± SEM).

**Phase**	**Item**	**T0**	**T1**	**T2**	**T3**	**T4**	**T5**	***P*-value**
The beginning	TC, mmol/L	2.88 ± 0.13	3.43 ± 0.14	3.47 ± 0.15	3.15 ± 0.2	3.13 ± 0.28	3.34 ± 0.29	0.228
(1–21 d)	TG, mmol/L	0.41 ± 0.06	0.37 ± 0.05	0.35 ± 0.04	0.34 ± 0.05	0.33 ± 0.02	0.32 ± 0.02	0.850
	TP, mmol/L	22.0 ± 0.37	23.0 ± 0.81	22.3 ± 0.98	23.6 ± 0.85	24.5 ± 0.96	23.1 ± 0.57	0.103
	ALB, g/L	14.7 ± 0.3	15.3 ± 0.34	16.6 ± 0.64	15.2 ± 0.51	15.7 ± 0.23	16.1 ± 0.55	0.148
	UA, μmol/L	489.0 ± 52.0	537.6 ± 68.7	649.6 ± 82.6	596.1 ± 52.5	589.3 ± 60.5	486.0 ± 71.4	0.59
The end	TC, mmol/L	3.41 ± 0.18	3.61 ± 0.2	3.71 ± 0.2	3.30 ± 0.2	3.20 ± 0.12	3.47 ± 0.13	0.216
(22–42 d)	TG, mmol/L	0.31 ± 0.01^b^	0.28 ± 0.02^ab^	0.27 ± 0.02^ab^	0.28 ± 0.01^ab^	0.24 ± 0.04^a^	0.26 ± 0.05^ab^	0.048
	TP, mmol/L	26.3 ± 1.16^a^	27.7 ± 1.44^ab^	26.9 ± 1.2^ab^	30.5 ± 2.29^ab^	32.2 ± 1.57^b^	29.8 ± 1.54^ab^	0.090
	ALB, g/L	14.7 ± 0.56	16.3 ± 1.09	16.4 ± 0.94	15.6 ± 0.65	15.63 ± 0.51	15.1 ± 0.75	0.062
	UA, μmol/L	116.2 ± 28.7^a^	156.8 ± 27.6^ab^	145.3 ± 30.4^ab^	235.3 ± 30.7^b^	211.6 ± 42.1^ab^	195.4 ± 32.4^ab^	0.019

a−d The same as the above table. TC, total cholesterol; TG, triglyceride; TP, total protein; ALB, albumin; and UA, uric acid.

**Table 5:** Effects of adding CAP difference level on antioxidant parameters of white-feather broilers (means ± SEM).

**Phase**	**Item**	**T0**	**T1**	**T2**	**T3**	**T4**	**T5**	***P*-value**
The beginning	GSH-PX (μmol/L)	33.9 ± 1.88^a^	34.4 ± 2.18^ab^	42.3 ± 2.8^ab^	47.7 ± 2.57^b^	47.7 ± 2.82^b^	43.4 ± 3.65^ab^	0.030
(1–21 d)	SOD (U/ml)	88.7 ± 2.36^a^	109.4 ± 6.7^ab^	120.1 ± 6.41^b^	118.4 ± 6.09^b^	107.2 ± 8.52^ab^	101.6 ± 7.41^ab^	<0.001
	MDA	0.65 ± 0.04^a^	1.21 ± 0.23^ab^	2.23 ± 0.18^b^	2.15 ± 0.09^ab^	1.69 ± 0.1^ab^	1.69 ± 0.14^ab^	0.023
	GSH/MDA	52.7 ± 2.58^b^	35.5 ± 6.33^ab^	19.7 ± 2.01^a^	22.4 ± 1.2^a^	28.1 ± 1.44^ab^	27.1 ± 3.24^ab^	<0.001
	SOD/MDA	138.6 ± 6.14^b^	139.4 ± 22.3^b^	55.2 ± 3.59^a^	55.4 ± 2.34^a^	58.9 ± 2.47^a^	58.6 ± 6.29^a^	<0.001
The end	GSH-PX(μmol/L)	40.0 ± 2.7^ab^	47.5 ± 2.23^b^	29.8 ± 3.00^a^	40.7 ± 2.19^ab^	41.7 ± 2.46^ab^	26.9 ± 2.84^a^	<0.001
(22–42 d)	SOD (U/ml)	76.5 ± 6.01^a^	112.6 ± 7.73^b^	86.7 ± 8.56^ab^	96.8 ± 6.96^ab^	103.8 ± 5.79^ab^	107.5 ± 7.79^ab^	0.024
	MDA	1.85 ± 0.22	1.91 ± 0.18	1.75 ± 0.14	1.72 ± 0.17	1.72 ± 0.10	1.93 ± 0.22	0.948
	GSH/MDA	23.6 ± 3.18^ab^	26.9 ± 3.39^b^	17.7 ± 2.43^a^	24.9 ± 2.46^ab^	24.6 ± 2.05^ab^	14.0 ± 1.24^a^	0.002
	SOD/MDA	40.4 ± 5.21	55.6 ± 4.77	53.3 ± 3.93	66.1 ± 8.36	66.3 ± 5.63	68.±7.96	0.023

a−d The same as the above table. SOD, activities and the enzymatic activities of superoxide dismutase; GSH-PX, glutathione Peroxidase; and MDA, malondialdehyde.

### Expression of lipid metabolism genes

**Figure 1:**
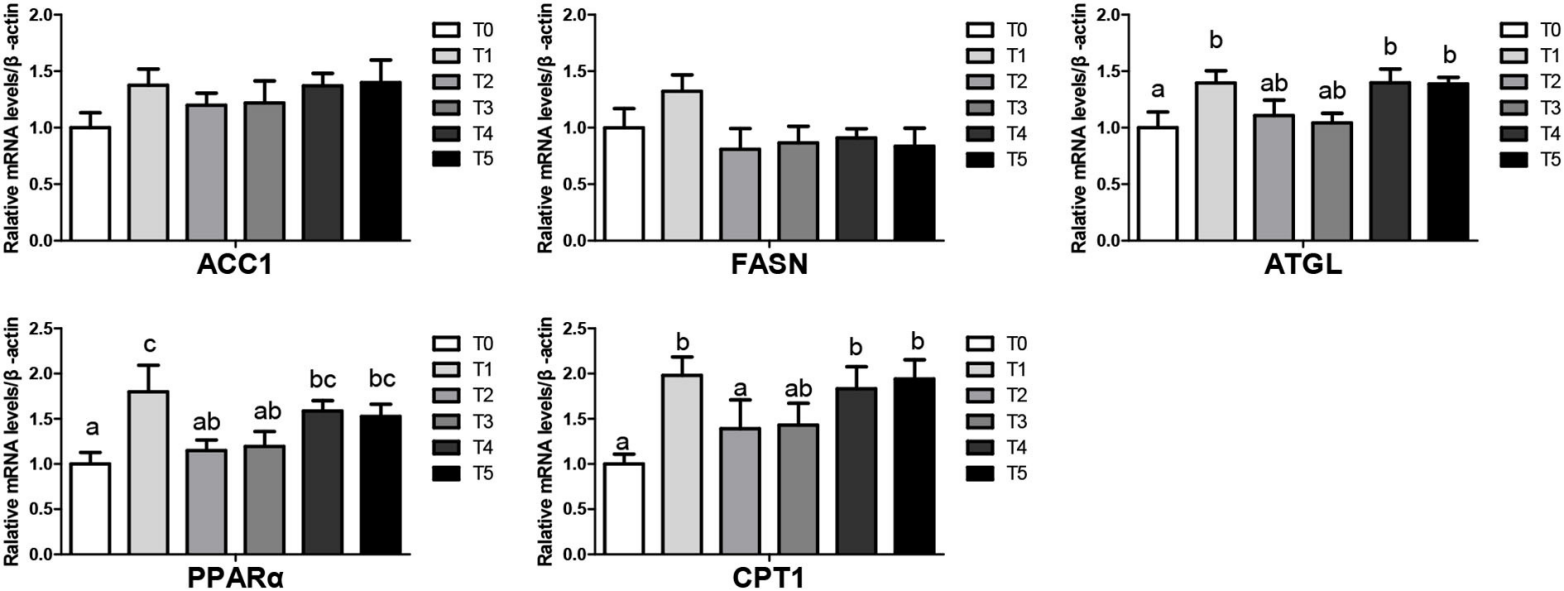
Lipid metabolism in the liver of broiler when CAP partially replaced dietary SBM at the beginning (values having different letters are significantly different, *P* < 0.05, mean s (means ± SEM). Calculated as thhase; ACC 1, acetyl-CoA carboxylase 1; CPT-1, Carnitine palmitoyltransferase; ATGL, adipose triglyceride lipase; PPAR, peroxisome proliferator-activated receptor.

**Figure 2:**
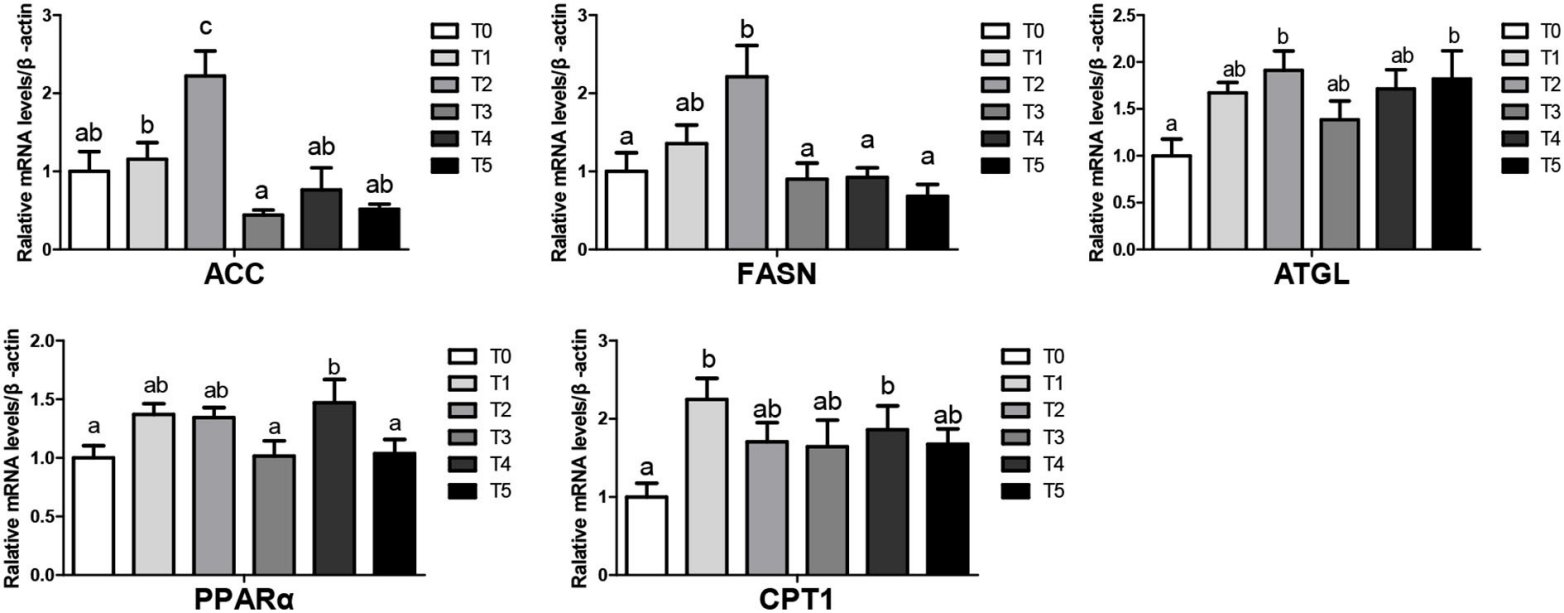
Lipid metabolism in the liver of broiler when CAP partially replaced dietary SBM at the end (values having different letters are significantly different, *P* < 0.05, mean activated receptor). Receptor. Ed assynthase; ACC 1, acetyl-CoA carboxylase 1; CPT-1, Carnitine palmitoyltransferase; ATGL, adipose triglyceride lipase; PPAR, peroxisome proliferator-activated receptor.

The mRNA levels of lipid metabolism-related genes in liver tissues at the beginning were shown in Figure 1. Broiler feed CAP improved lipolysis genes (ATGL, PPARα, CPT1) and gene mRNA levels in T1, T4, T5 groups (*P* < 0.05). There was no significantly different in lipogenesis (ACC1, FASN) mRNA level (*P* > 0.05). In the end, The mRNA levels of lipid metabolism-related genes in liver tissues were shown in Figure 2, Upregulated lipogenic genes (ACC1, FASN) of T2 were significantly increased (*P* < 0.05). ATGL and PPAR of T2, T4, and T5 were increased (genes in T4 and T5 were significantly higher than T0 (*P* < 0.05).

### Hepatic histological examination

**Figure 3:**
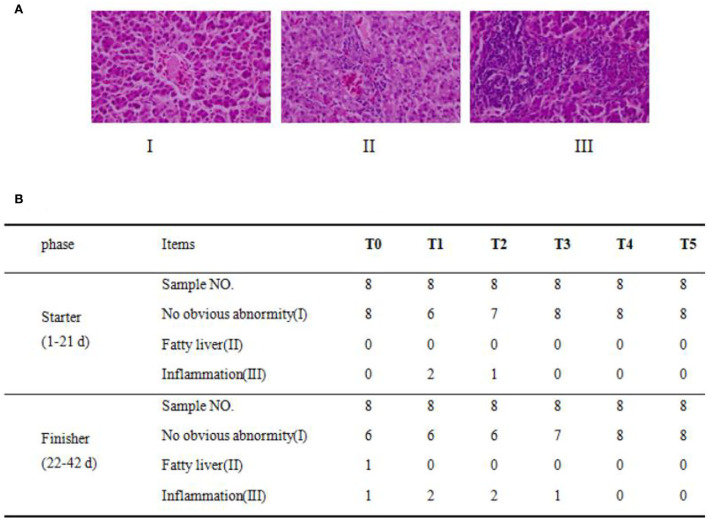
Dietary CAP induced liver histological lesions and increased inflammation. **(A)** Histopathological analysis of broiler. Three phenotypes of hepatic histopathological examination with symptoms from light to heavy by HandE staining (*n* = 8) under a magnification of 400×, in which there were (I): no obvious abnormity, (II): fatty liver or steatohepatitis, (III): inflammatory cell infiltration, and **(B)** Phenotypes statistical results (*n* = 8).

Broiler liver sections were examined after H&E staining for collagen, and eight samples were observed in each group. In Figure 3A, three typical phenotypes are observed on the hepatic samples of the broiler. Phenotype I showed normal hepatocytes with well-shaped cells and evenly distributed cytoplasm. Phenotype II defined the structure of the hepatic lobule was unclear. Part of the hepatic cord structure disappeared, the arrangement of liver cells was disordered, the liver became narrow, and there were many lipid-like vacuoles in liver cells. Phenotype III defines inflammation as a large number of inflammatory cell infiltration in the liver tissue, hepatocyte degeneration, necrosis, and widespread necrosis. The hepatic histopathological examination results of each group are shown in Figure 3B. All treatment groups showed more liver damage than the T0 group. At the beginning in the T0 group, there were no phenotypes II and III and eight with normal (phenotype I). All dietary CAP inclusion groups showed no serious damage to hepatic health. In the T1 group, only two samples were typically inflamed (phenotype III), there was no phenotype II, and six samples were considered normal (phenotype I). In the T2 group, only one sample was observed as being inflamed (phenotype III), there were no phenotype II samples, and seven samples showed no obvious abnormity (phenotype I). All samples showed a normal morphological structure in the T3, T4, and T5 groups.

In the end, in the T0 group, there was only one sample with inflammation (phenotype III), one with phenotype II, and six with normal phenotypes (phenotype I). Six samples showed normal morphological structure in the T1 and T2 groups, and two exhibited inflammation. In the T3 group, only one sample was observed as inflammation (phenotype III), and seven with no obvious abnormity (phenotype I). In T4 and T5 groups, all samples showed a normal morphological structure. In general, dietary CAP inclusion will not cause the occurrence of inflammation.

### Composition and association analysis of gut microbiota

**Figure 4:**
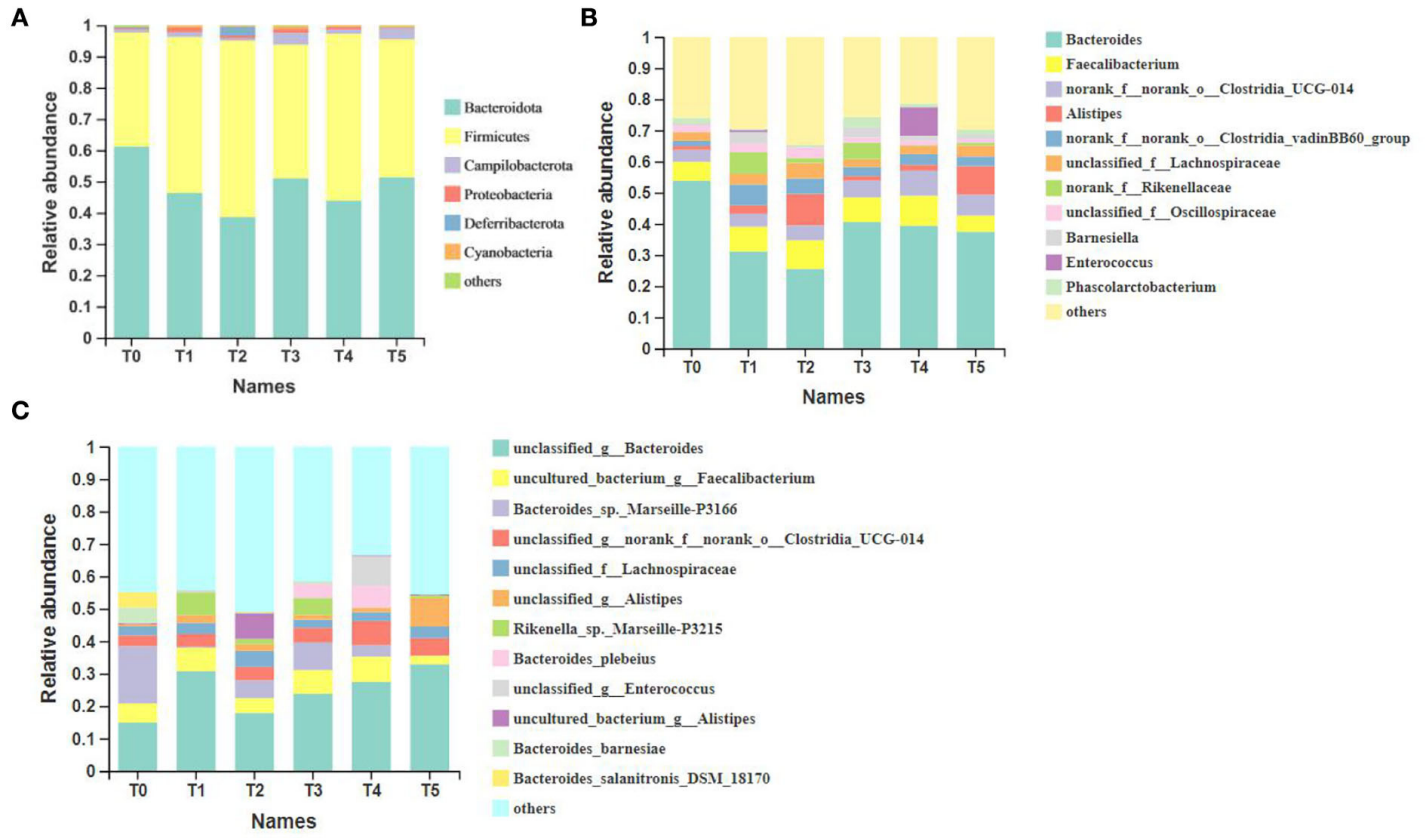
All taxa at three different levels and the top more than ten most abundant taxa of all samples (*n* = 6). **(A)** Phylum; **(B)** genus; **(C)** species. o__: order; f__: family; g__: genus. T0: fed diets based on SBM as the only protein source; T1, T2, T3, T4, T5: 1, 2, 3, 4, and 5% of CAP were added.

The taxonomic composition in Figure 4 was analyzed at the level of phylum, genus, and species. At the phylum level, the major taxa in all groups were *Bacteroidota, Firmicutes*, and *Campilobacterota*, and the proportion of the first two types of microbiota exceeded 35%. Compared to the T0 group, *Bacteroidota* in treatment groups decreased, while *Firmicutes* increased. At the genus level, the top ten microbiota of dominant groups were *Bacteroides, Faecalibacterium, o__Clostridia_UCG-014, Alistipes, o__Clostridia_vadinBB60_group, f__Lachnospiraceae, f__Rikenellaceae, f__Oscillospiraceae, UCG-005* and *Barnesiella*. The proportion of *Bacteroides* showed a decrease compared to control treatments, while the proportion of *o__Clostridia_UCG-014, o__Clostridia_vadinBB60_group*, and *f__Rikenellaceae* showed overall induction. Except for the predominant microbiota above, the proportion of *Enterococcus* increased significantly in the T4 group (*P* < 0.05). At the species level, the top ten microbiota of dominant groups were *g__Bacteroides, g__Faecalibacterium, Bacteroides_sp._Marseille-P3166, o__Clostridia_UCG-014, f__Lachnospiraceae, g__Alistipes, Rikenella_sp._Marseille-P3215, f__Oscillospiraceae, o__Clostridia_vadinBB60_group*, and *Bacteroides_plebeius*. The proportion of *Bacteroides_sp._Marseille-P3166* showed decreased trends compared to control treatments, while the proportion of *g__Bacteroides, g__Faecalibacterium, g__Alistipes, Rikenella_sp._Marseille-P3215* and *Bacteroides_plebeius* showed an overall induction.

**Figure 5:**
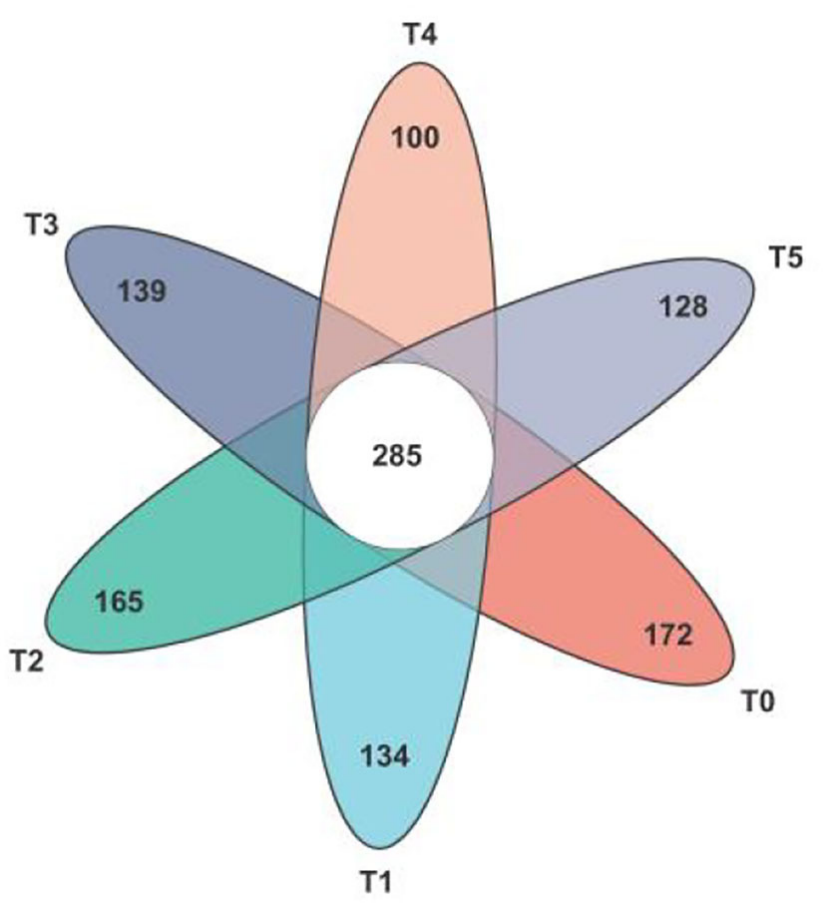
Venn diagram of the gut microbiota composition in Cobb broilers shows the number of shared and unique core ASVs among the six study groups. T0: fed diets based on SBM as the only protein source; T1, T2, T3, T4, T5: 1, 2, 3, 4, and 5% of CAP were added.

In total 1990 ASVs, 1,123 ASVs were identified as core ASVs according to their prevalence in each sample. In Figure 5, a total of 285 ASVs were universally present in all 36 samples, of which 91% were classified as *Firmicutes*, 7% were classified as *Bacteroidota*, and after classifying the above ASVs at the species level, the top five microbiota were *o__Clostridia_UCG-014, f__Lachnospiraceae, f__Oscillospiraceae, g__Bacteroides* and *o__Clostridia_vadinBB60_group*. Besides the T2 group, the other groups were lower than the number of unique ASVs in the T0 group.

**Figure 6:**
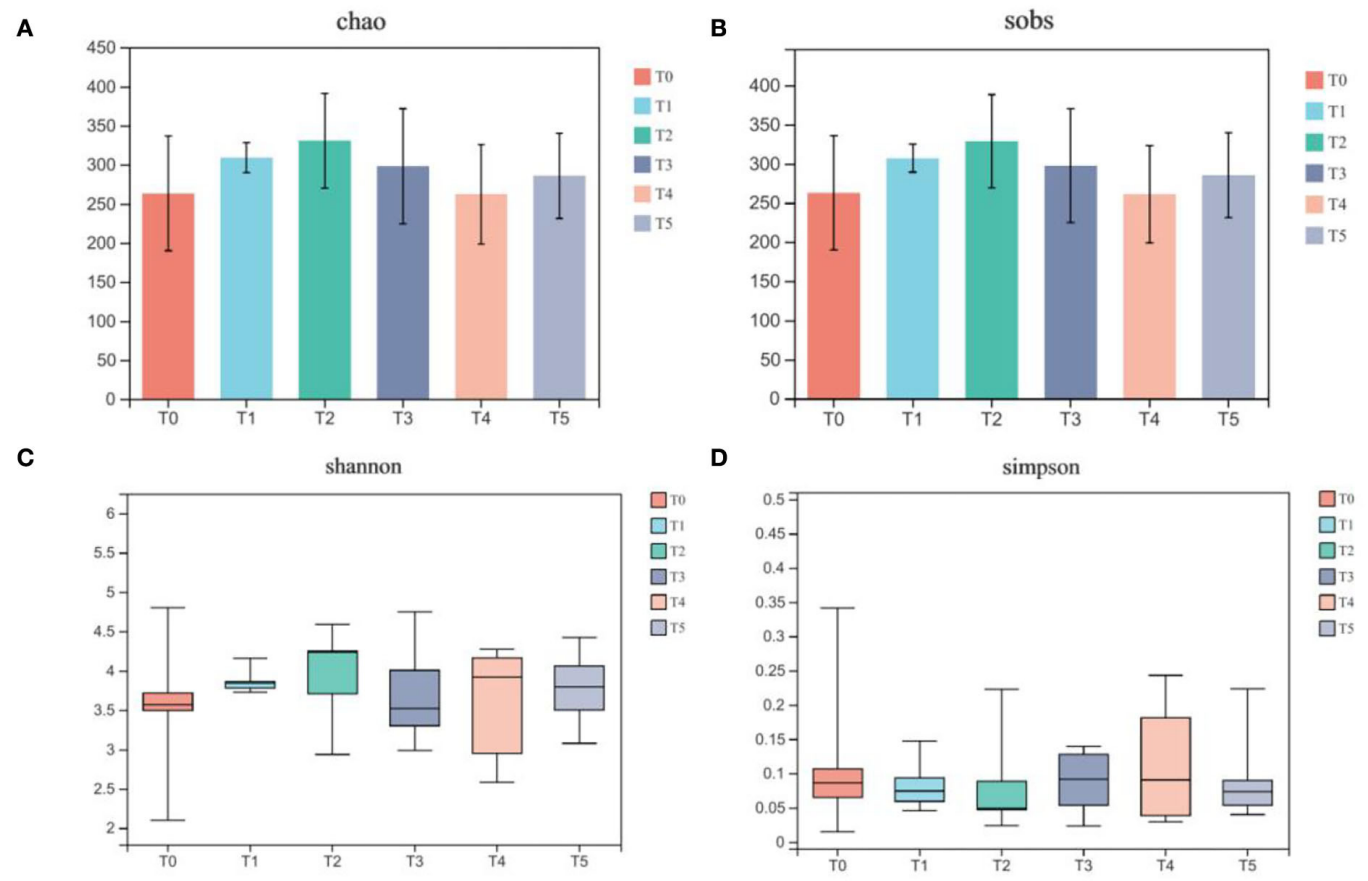
Dietary supplementation of CAP affects the alpha diversity of gut microbiota in Cobb broilers (*n* = 6). **(A)** Chao; **(B)** sobs; **(C)** Shannon; **(D)** Simpson. Multiple comparisons were determined by one-way analysis of varlance (one-way ANOVA), followed by Tukey-Kramer as a *post hoc* test. T0: fed diets based on SBM as the only protein source; T1, T2, T3, T4, T5: 1, 2, 3, 4, and 5% of CAP were added.

In Figure 6, the alpha-diversity indices relating to community richness, including sobs and Chao, presented the tendency to increase first and then decrease. No significant differences between the above two indexes were found between control and treatment groups (*P* > 0.05), although the index value in treatment groups was higher than in control groups. For Shannon and Simpson, which relates to community diversity, the dispersion degrees were relatively increased, but with no significant difference in index values (*P* > 0.05).

Cobb broilers' intestinal SCFAs, plasma SCFAs, and digesta bile acids are displayed in Tables 6–8, which contain the related microbiome metadata. P 0.05 denoted a statistically significant difference determined using Duncan's multiple range test. In the intestine, the content of acetic acid and propionic acid was significantly increased by CAP addition (*P* < 0.05). At the same time, with the increase of the additional quantities, the content of butyric acid, valeric acid, isobutyric acid, and isovaleric acid was higher in the treatment groups than in the control group. Additionally, in plasma, the addition of CAP caused a significant increase in the content of acetic acid, propionic acid, butyric acid, and isobutyric acid (*P* < 0.05). Regarding bile acids, the findings show that CAP addition led to a statistically significant increase in primary bile acids such as cholic acid (CA) and chenodeoxycholic acid (CDCA), but for secondary bile acids like taurocholic acid (TCA) and taurochenodeoxycholic acid (TCDCA), it behaved a general trend of first increase and then decreased.

**Table 6:** Effects of adding CAP level (μg/L) on SCFAs in the intestine of white-feather broilers (means ± SEM).

**SCFAs**	**T0**	**T1**	**T2**	**T3**	**T4**	**T5**
Acetic acid	1,438.04 ± 158^a^	2,846.7 ± 171.4^b^	8,471.72 ± 509^d^	7,996.79 ± 126^d^	7,572.87 ± 654^d^	5,299.05 ± 185^c^
Propionic acid	662.89 ± 152.7^a^	2,641.23 ± 265^b^	6,944.05 ± 584^d^	8,856.64 ± 376^e^	6,743.73 ± 556^d^	5,209.38 ± 207^c^
Butyric acid	611.84 ± 84.6^a^	1,076.35 ± 131^a^	5,451.67 ± 470^c^	5,164 ± 360.5^c^	4,513.66 ± 532^c^	2,885.65 ± 185^b^
Valeric acid	403.9 ± 45.8^a^	841.64 ± 102.7^a^	2,438.29 ± 140^c^	2,610.55 ± 150^c^	1,961.59 ± 289^b^	1,891.92 ± 62.1^b^
Isobutyric acid	360.95 ± 52.8^a^	585.49 ± 72^a^	1,506.38 ± 80.8^b^	1,984.23 ± 115^c^	2,011.35 ± 94.8^c^	1,344.6 ± 15.7^b^
Isovaleric acid	262.46 ± 90.1^a^	570.58 ± 110^ab^	1,147.96 ± 116^c^	1,807.22 ± 210^d^	1,135.34 ± 34.7^c^	931.17 ± 137^bc^

a−e Indicate a significant difference (at the *P* < 0.05).

**Table 7:** Effects of adding CAP level (μg/L) on SCFAs in the plasma of white-feather broilers (means ± SEM).

**SCFAs**	**T0**	**T1**	**T2**	**T3**	**T4**	**T5**
Acetic acid	1,667.7 ± 31^a^	2,040 ± 54^b^	2,118.4 ± 44^b^	2,217 ± 32^bc^	2,337.3 ± 91^c^	2,102.5 ± 32^b^
Propionic acid	300.35 ± 21^a^	371.3 ± 13^b^	379.6 ± 30^b^	477.3 ± 22.4^c^	570.9 ± 26.4^d^	387.3 ± 35.6^b^
Butyric acid	345.4 ± 14.2^a^	456.4 ± 28.9^b^	461.6 ± 35.6^b^	640.6 ± 41.0^c^	441.8 ± 22.7^b^	379.9 ± 16.7^a^
Valeric acid	69.38 ± 4.1d^e^	50.15 ± 2.1^ab^	62.77 ± 3.5^cd^	77.55 ± 4.03^e^	47.7 ± 2.6^a^	57.6 ± 1.4^bc^
Isobutyric acid	113 ± 4.63^a^	147.7 ± 4.76^bc^	158.43 ± 4.61^c^	183.42 ± 7.48^d^	177.14 ± 5.22^d^	132.46 ± 9.83^b^
Isovaleric acid	48.51 ± 2.6^a^	63.11 ± 2.69^bc^	56.42 ± 2.84^b^	64.37 ± 1.7^c^	56.5 ± 1.73^b^	44.18 ± 2.39^a^

a−e Indicate a significant difference (at the *P* < 0.05).

**Table 8:** Effects of adding CAP level (ng/mg) on bile acids in the digesta of white-feather broilers (means ± SEM).

**Bile acids**	**T0**	**T1**	**T2**	**T3**	**T4**	**T5**
Cholic acid	14.94 ± 7^a^	46.14 ± 51.1^c^	45.79 ± 54.3^c^	43.99 ± 39.4^c^	20.58 ± 18.6^b^	71.80 ± 84.9^d^
Taurocholic acid	167.01 ± 140.7^d^	107.25 ± 129.3^a^	108.99 ± 112.3^a^	133.03 ± 154.7^c^	125.82 ± 139.2^b^	222.90 ± 284.9^e^
Taurochenodeoxycholic acid	70.83 ± 59.5^d^	52.82 ± 68.8^b^	49.20 ± 68^b^	40.99 ± 38.9^a^	41.89 ± 43.9^a^	57.74 ± 71.6^c^
Chenodeoxycholic acid	41.74 ± 20.7^a^	76.90 ± 39.2^b^	87.21 ± 66.1^c^	107.01 ± 60.8^d^	44.35 ± 48^a^	90.16 ± 142.1^c^

a−e Indicate a significant difference (at the *P* < 0.05).

**Figure 7:**
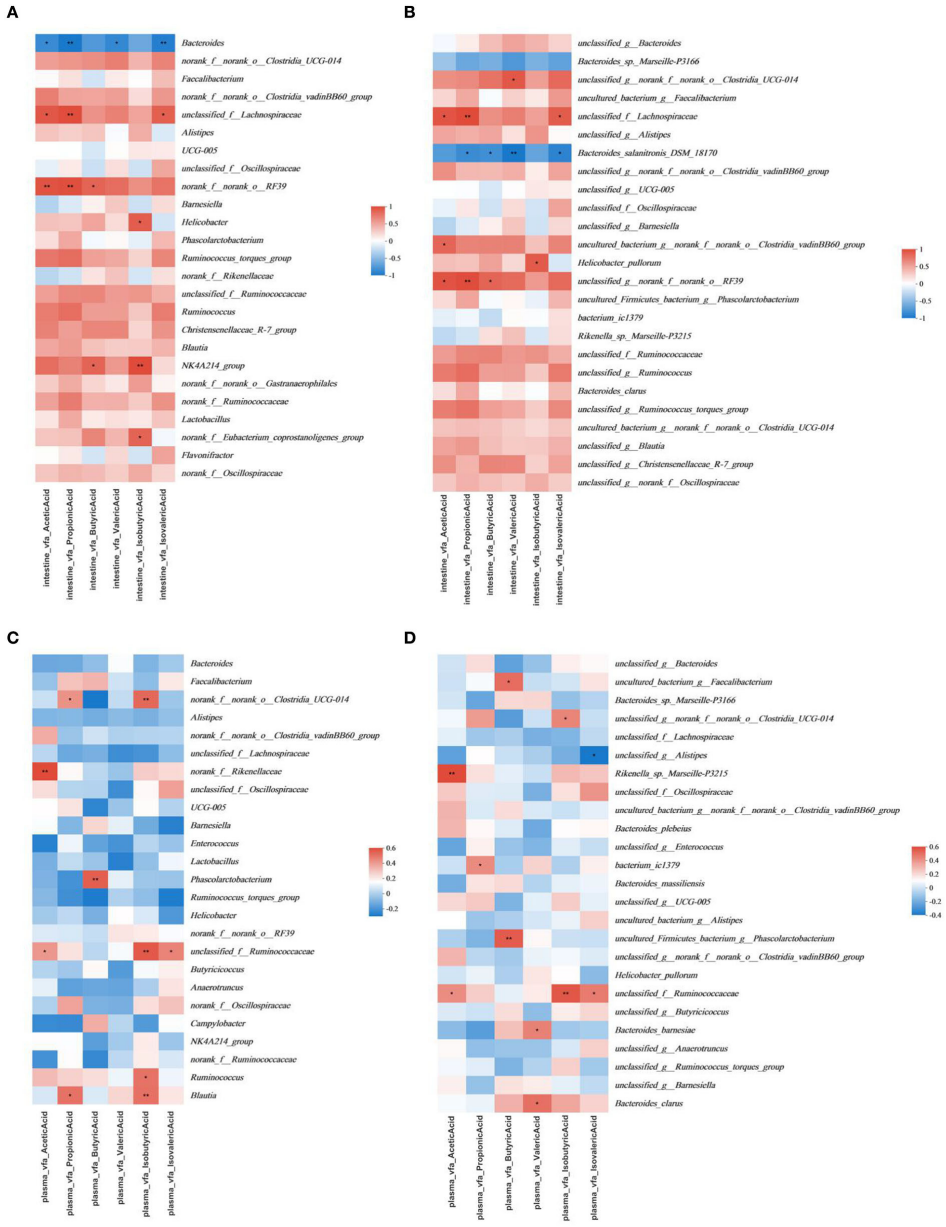
Significant associations between intestinal and plasma microbial and sample metadata of intestinal SCFAs (*n* = 6). **(A)** Intestinal in the genus; **(B)** intestinal in species; **(C)** plasma in the genus; **(D)** plasma in species. Cells that denote associations are colored (red or blue) and overlaid with asterisks which show statistically significant differences (*, *P* < 0.05; **, *P* < 0.01; ***, *P* < 0.001). o__: order; f__: family; g__: genus. T0: fed diets based on SBM as the only protein source; T1, T2, T3, T4, T5: 1, 2, 3, 4, and 5% of CAP were added.

**Figure 8:**
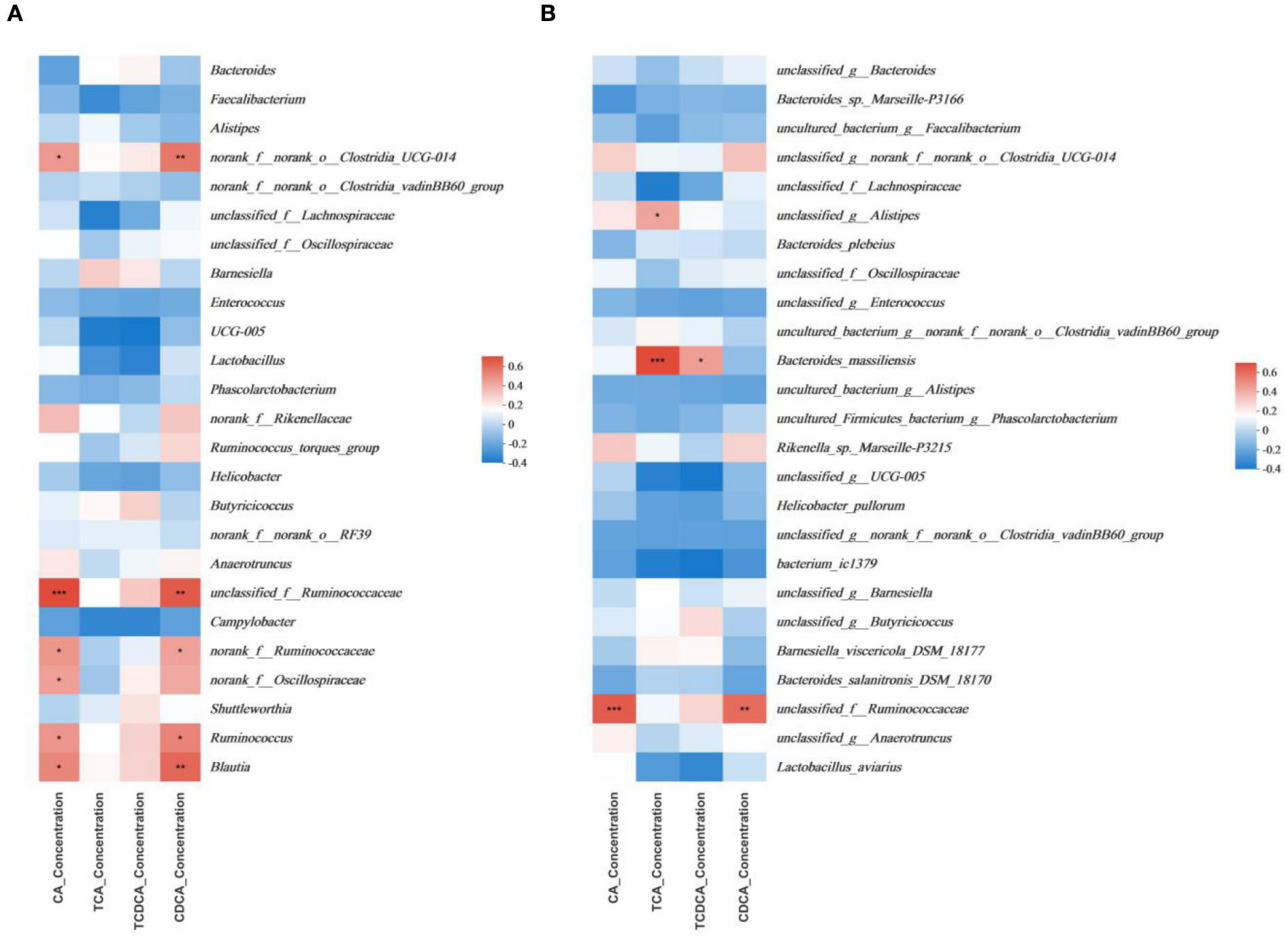
Significant associations between intestinal microbial and sample metadata of digesta bile acids (*n* = 6). **(A)** genus; **(B)** species. Cells that denote associations are colored (red or blue) and overlaid with asterisks which show statistically significant differences (*, *P* < 0.05; **, *P* < 0.01; ***, *P* < 0.001). o__: order; f__: family; g__: genus. T0: fed diets based on SBM as the only protein source; T1, T2, T3, T4, T5: 1, 2, 3, 4, and 5% of CAP were added.

The multivariate association analysis in Figures 7, 8 was conducted on the genus and species levels. According to the metadata source, we analyzed the top 25 microbiota in terms of total abundance at various taxonomic levels. The multivariate association analysis of intestinal SCFAs in Cobb broilers at genus and species levels showed that eight microbiota, including *Helicobacter_pullorum, o__Clostridia_UCG-014, f__Lachnospiraceae* and *o_RF39*, were positively correlated with all the six short-chain acids (*P* < 0.05). *Bacteroides* and *Bacteroides_salanitronis_DSM_18170* were negatively correlated with the other five short-chain acids, with the exception of isobutyric acid (*P* < 0.05). Meanwhile, the Analysis of plasma SCFAs in Cobb broilers at the above two levels showed that 12 microbiota, including *Rikenella_sp._Marseille-P3215*, g__Phascolarctobacterium, Bacteroides_barnesiae, Bacteroides_clarus, *o__Clostridia_UCG-014*, and *f__Ruminococcaceae*, were positively correlated with all the six short-chain acids (*P* < 0.05), and *g__Alistipe* was negatively correlated with isovaleric acid (*P* < 0.05). Finally, the analysis of digesta bile acids in Cobb broilers also at the above two levels showed that eight microbiota, including *g__Alistipes, Bacteroides_massiliensis*, and *f__Ruminococcaceae*, were positively correlated with all the four bile acids (*P* < 0.05). Furthermore, it can also be determined that four microbiota, including *Blautia, Ruminococcus, o__Clostridia_UCG-014*, and *g__Alistipes*, were significantly correlated with both some SCFAs in plasma and some bile acids in digesta (*P* < 0.05).

## Discussion

### Growth performance effect

Several functions of CAP have been demonstrated, such as promoting growth performance and improving immune ability in carp and Tilapia mossambica (31). Some researchers found that CAP has no apparent negative impact on growth performance in a particular proportion. With the increase of CAP, WG and SGR initially increased and then decreased, and the peak value appeared in the CAP 14.55% diet (22). Using 5% CAP to replace 27.6% SBM was helpful to the growth performance of Grass Carp, according to the results of a trial conducted by other researchers (19). Some researchers have explored using CAP as an effective protein source for fish, but there is little research on poultry. In the poultry industry, it takes at least 42 days for a broiler to reach market size. Compared to the control group, which was fed only SBM, feeding 2.5 or 5% SCP resulted in greater body weight growth over various phases and cumulatively (32). Additionally, our study showed improvements in ADFI in the beginning phase when CAP was included in Cobb broiler diets. Therefore, for BWG, there were significantly increased values in the T2 group, consistent with the above literature findings, showing the improvements in production performance when CAP was included in Cobb broiler diets.

Moreover, the effects of F/G quality could significantly reflect the broiler's growth. As some researchers have explored, the feed conversion efficiency is greatly influenced by feed digestibility (33, 34). In our present study, CAP effectively reduced F/G at the beginning and overall phases, and when adding 4% CAP, the feed conversion efficiency was highest, which meant broilers had better feed conversion. Other research results have also confirmed these points.

### Protein metabolism and fat deposition effect

TP and ALB can reflect the body's nutritional status and protein metabolism. Additionally, the content increases with the increase in nutrient absorption (35–37). UA is an indicator of amino acid utilization and nitrogen-containing waste after protein decomposition (38–40). In the present study, we found that adding CAP to the diet could effectively improve the content of TP in broiler plasma at the last phase. Thus, this change indicated that feeding with CAP could increase protein absorption while improving growth performance (41). The blood UA level changed with the increasing CAP addition during the whole experiment. The broiler absorbed CAP more easily than the soybean, but more nitrogen content was excreted from the body. CAP could increase BW and decrease fat deposition in broiler chickens. In some articles, reducing abdominal fat or fat deposition is accompanied by improved growth performance or a better material-weight ratio (41, 42). There is no clear conclusion, but combined with experience, CAP can reduce abdominal fat deposition and improve feed conversion efficiency.

### Lipid metabolism genes effect

Adipose tissue formation is a complex process affected by many alterations in the expression of lipogenesis and lipolysis genes. ACC1 catalysis acetyl-CoA to malonyl-CoA is the first step for fatty acid synthesis and limits fatty acid biosynthesis (43). FASN is the key enzyme for the de novo synthesis of fat (44). The synthesis and decomposition of fat are carried out simultaneously. Inhibiting lipogenesis and de novo lipolysis can reduce fat storage by regulating the expression of lipogenic genes (45, 46). Our research showed no significant changes in ACC1 and FASN at the beginning or the end, except for T2. So, the lipid synthesis genes of broilers treated with CAP remained stable.

ATGL is the most important lipase for initiating triglyceride hydrolysis and does not require hormone activation (47). PPARα participates in the transcriptional regulation of lipid metabolism and liver fatty acid oxidation to reduce fatty acid synthesis (48, 49). ATGL can promote fatty acid oxidation by increasing the activity of PPARl (50, 51). CPT-1 is the key gene that controls mitochondrial and beta-oxidation of fatty acids (52, 53), which can increase the rate of lipolysis and activity of lipolytic enzymes (54). Our results showed a noticeable increase in ATGL, PPAR, and CPT-1. It could reduce the levels of TG (55, 56). TG is the main form of nutrients in the blood after digestion and absorption by the body, which can continuously accumulate to form fat. Comprehensive results: in treatment groups, the content of TG was reduced by upregulated lipolysis gene's expression level, leading to fat deposition reduction.

### Antioxidant capacity effect

Lipid metabolism is known to relate to oxidation reaction (57) and oxidation reaction reduction of fat (58, 59). A deficiency of ATGL and lipolytic genes may cause oxidative stress and inflammation and even affect organ health (60). CPT1 can protect hepatic mitochondria function and attenuate oxidative stress by activating the Nrf2 pathway (61, 62). Antioxidant capacity affects poultry health, which is reflected in SOD, GSH-Px, and MDA. SOD can reflect the ability to scavenge free radicals and promote the synthesis of immune-related proteins (63). Free radicals produce oxygen molecules and hydrogen peroxide under the action of SOD. CAT catalyzes the decomposition of hydrogen peroxide, thus reducing lipid superoxide damage and scavenging free radicals (64).

MDA is the final product of lipid peroxidation and can be used to measure oxidative damage (65). In the present study, SOD and MDA increased with the addition of CAP in the beginning phase. The change of SOD/MDA resulted from the common change of SOD and MDA. Both values increased, but the decrease of the SOD/MDA indicates that MDA increases more. Thus, we speculated that MDA played a leading role in both changes. It may be that adding CAP to increase the content of MDA will increase the activity of GSH-Px and SOD within the adjustment range or other reasons, which require follow-up research. In the last phase, with the addition of CAP, SOD increased, increasing SOD/MDA, while MDA returned to normal in all groups. This may be because CAP can improve antioxidant enzyme activity, effectively remove excessive free radicals, and prevent lipid peroxidation-related damage. In a similar paper (66), the antioxidant index of T-AOC was caused by increased breeder dietary vitamin E, thereby reducing the MDA level. In the article on CAP, the addition and use of CAP improve the antioxidant capacity of fish. Thus, CAP generally improves the antioxidant capacity of broilers. It may come into force at the last stages. It may be due to individual differences. In this study, the phenotype of animals did not change linearly in the same proportion as the additive proportion. But all of the changes were toward positive phenotypes.

### Liver histology effect

The liver is the main organ of metabolism and protein synthesis (67). In addition to the T4 group, the amount of liver inflammation increased slightly by adding CAP to the diet. This may be due to excessive pressure on metabolism or detoxification (68). Because of intestinal health or changes in gut microbiota and liver inflammation (69). Because this is the first study of CAP in the field of broilers, the conclusions on the use of CAP in broilers need to be updated according to the follow-up research. Further research can focus on intestinal microbial composition, intestinal reabsorption, and blood composition.

### Core microbiota effect

*Chickense* is the first study of CAP in the field of broilers. The conclusions on using CAP in broilers need to be updated according to the follow-up research. The gut microbiota is in a symbiotic relationship with its hosts and stays in a stable state for a long time. When the abundance and diversity of microbiota in the gut are at high levels, its own microbiota has a strong competitive power while reducing the overgrowth of foreign microbiota and enhancing immunity. Among the alpha-diversity indices, the Chao and sobs indexes are used to evaluate the abundance of a microbial community, and their values mainly reflect the number of species in the environment. The Shannon and Simpson indexes are mainly used to measure the diversity and uniformity of the microbial community. In the present study, we found that the treatment groups' Chao, sobs, and Shannon indices all have a little increase, and the fluctuation of the Shannon indices also increases. Thus, this change indicated that the addition of CAP could not only increase the number of gut microbiota but also improve the richness and evenness of the gut microbiota to a certain extent.

Many studies have shown that the *Firmicutes* and the *Bacteroidetes* are the dominant microbiota in the mammalian gut, accounting for more than 90% of the population (70). *Firmicutes/Bacteroidetes* show a positive association with body weight in a certain range (70). Other studies have proved that when the *Firmicutes/Bacteroidete* ratio is high, it will have a strong fermentation ability and can produce more SCFAs for better nutrient absorption (71). In the present study, of the ASVs prevalent in all samples, 91% were classified as *Firmicutes*, and 7% were classified as *Bacteroidetes*, which were higher than other microbiota, indicating that *Firmicutes* and *Bacteroidetes* were the dominant microbiota in the intestinal tract of Cobb broilers. Meanwhile, in the taxonomical composition analysis at the phylum level, the addition of CAP decreased the abundance of *Bacteroidetes*. It increased the abundance of Firmicutes in broiler cecum microorganisms compared with the control group. As can be seen, adding CAP can increase *Firmicutes/Bacteroidetes* and improve the growth performance of Cobb broilers to some extent.

### Significant interactions SCFAs and gut microbiota

SCFAs are metabolites produced from fiber fermentation by intestinal microbiota (72). SCFAs contribute to the absorption and utilization of nutrients, and they can be used as energy substrates and positively influence growth. They also affect digestion and absorption of nutrients by altering intestinal pH and further affect the body's metabolic activity. SCFAs include acetic acid, propionic acid, butyric acid, valeric acid, isobutyric acid, isovaleric acid, and so on. Among them, *Bacteroidetes* mainly produce acetic and propionic acids, while butyrate is mainly produced by *Firmicutes* (73). *Family Ruminococcaceae*, species *Bacteroides uniformis* and *Bacteroides ovatus* are all degraders of plant polysaccharides such as cellulose and hemicellulose, which may lead to the production of SCFAs (74). In the intestinal epithelial cells specific aryl hydrocarbon receptor deficiency (AhrDeltaIEC) mice, not only was the overgrowth of *H. hepaticus* and *H. ganmani* detected in the gut, but it was also found that among the altered metabolites, the amount of isobutyric acid in the cecal of AhrDeltaIEC mice was increased compared to the control group (75). *Phascolarctobacterium* has been identified as butyric acid-producing gut microbiota in humans (76). In animal experiments, the increase in the relative abundance of SCFAs, including acetic acid, propionic acid, and butyric acid, was detected in the intestines of piglets treated with Gegen Qinlian decoction (GQD) for infectious diarrhea. The same increase was detected in the relative abundance of microbiota, including *Phascolarctobacterium* (77). Moreover, as microbiota isolated from the broiler intestine, there is substantial variation between *Bacteroides_barnesiae* and *Bacteroides_clarus* in the adaptability to broilers (78, 79).

In the present study, we found that in the multivariate association analysis on the intestine, the decreasing trend of *Bacteroides* was consistent with the increasing trend of SCFAs in treatment groups of the cecum. At the species level, *Bacteroides_sp._Marseille-P3166* and *Bacteroides_salanitronis_DSM_18170* were negatively correlated with all SCFAs. They negatively correlated significantly with propionic acid, butyric acid, valeric acid, and isovaleric acid. That is to say, the tendency of microbiota was consistent with the change of the four SCFAs above. Combined with the *p-*values, it can be inferred that the above two *Bacteroides* are the main factors affecting the correlation between *Bacteroides* and six SCFAs above in the gut of cobb broilers, and *Bacteroides_salanitronis_DSM_18170* had a much stronger correlation with the contents of above four SCFAs compared to *Bacteroides_sp._Marseille-P3166*. The proportion of *Helicobacter_pullorum* and the mean content of isobutyric acid increased in the T3 and T5 test groups, and the proportion of *Helicobacter_pullorum* changed in the same trend as Helicobacter. Therefore, it can be assumed that *Helicobacter_pullorum* is the main factor affecting the correlation between *Helicobacter* and isobutyric acid.

Other correlations exist in the multivariate association analysis based on plasma SCFAs. The increasing trend of *Phascolarctobacterium* was consistent with that of butyric acid in the treatment group. At the species level, the trend for proportions of *g_Phascolarctobacterium* was consistent with *Phascolarctobacterium*, so it can be assumed that the proportion of *g_Phascolarctobacterium* was the main factor affecting the correlation between *Phascolarctobacterium* and butyric acid, and could account for the decrease of butyric acid content in the T4 and T5 treatment groups which similar to that in T0 control group. The proportion of *Bacteroides_barnesiae* continued to decrease in the treatment group, and the proportion of *Bacteroides_clarus* showed an increase in the T3 treatment groups, while the average content of valeric acid in the plasma of treatment groups was lower than that of the control group, and there was a peak value in the T3 treatment group. Combined with the *p*-value, it could be inferred that the content of valeric acid in the plasma of Cobb broilers was indeed affected by the *Bacteroides_barnesiae* and *Bacteroides_clarus*. In addition, *Bacteroides_sp._Marseille-P3166* and *Rikenella_sp._Marseille-P3215* had opposite correlations with acetic acid, the former was positively correlated with the content of acetic acid in plasma, and the latter was negatively correlated with it in the cecum. Given the existence of the same *sp*. classification level, these microbiotas could be further studied. All results in this study were consistent with the above literature findings.

### Significant interactions between bile acids and gut microbiota

Bile acids, the sterol of cholesterol metabolism in animals, are the main constituents of bile. They are synthesized in the liver, secreted into bile, and then excreted into the duodenum. They can undergo bacterial biotransformations in the gut and reabsorb into the blood. They are shown to be the main natural forcing factors and play a role in hepatic fat metabolism in the liver. Bile acids in the gut have been associated with bacterial overgrowth, while gut microbiota metabolizes bile acids to regulate the hosted metabolism (80). Bile acids are categorized into primary and secondary bile acids, hepatocytes synthesize the former, and the latter is converted primary bile acids into secondary bile acids by gut microbiota in the distal gut. Thus secondary bile acids are more susceptible to the gut microbiota (81). Research results indicated that when clostridia were associated only with GF (germ-free) mice, free-form bile acids accounted for less than 40% of total bile acids, and the percentage of secondary bile acids was similar to that of the other groups (82). The blue light treatment significantly increased the beta diversity of gut microbiota and the relative abundances of bacteria-producing bile acid hydrolase, especially *Alistipes*. These changes promoted the synthesis of secondary bile acids (83). In experiments about human feces, one of the secondary bile acid-producing bacteria was identified as *Bacteroides Intestinalis AM-1*, which could convert CA and deoxycholic acid (TCDA) into 7-oxo-DCA, 7-oxo-cholic acid, and other secondary bile acids (84).

There is a negative correlation between *Ruminococcaceae_NK4A214_group* and bile acid content, which leads to vitamin A deficiency in the intestinal tract (85). In the present study, we found that in the multivariate association analysis on digesta, *o__Clostridia_UCG-014* was not highly correlated with TCA and TCDCA and had no influence on the content of secondary bile acids. However, it showed significant positive correlations with primary bile acids such as CDCA and CA, so the correlation remains to be further studied. Also, *g__Alistipes* and *Bacteroides_massiliensis* both had significant positive correlations with TCA, the correlation of the latter is lower compared to the former, and the changing trend of TCA content was consistent with that of the above two microbiota. Therefore, we have come to the preliminary conclusion that both g__Alistipes and Bacteroides_massiliensis could determine the average content of TCA. Furthermore, the changes in the consent of CA and CDCA were consistent with the changes in the proportion of *f __Ruminococcaceae*, so the correlations with primary bile acids need further consideration. These above findings were consistent with the above literature findings.

## Conclusion

In conclusion, CAP can reduce the feed-to-gain ratio, promote protein metabolism markers and feed conversion rate, decrease abdominal fat yield, and effectively improve the antioxidant capacity in the final period. Meanwhile, CAP can increase the content of SCFAs and primary bile acids in the intestine and plasma, increase the *Firmicutes/Bacteroides* value, upregulate the abundance of beneficial bacteria, and elucidate the microbial mechanism of CAP on improving growth performance in Cobb broilers. So CAP has potential in Cobb broiler feed. It is recommended to add 4% CAP for promoting growth and maintaining the intestinal health of Cobb broilers. Subsequently, more experiments are still needed to verify the existing results and explore the deeper mechanism to ensure that the use and dosage of CAP in the future are more explicit, which can be popularized and applied in broiler farming after ensuring safety and effectiveness.

## Data availability statement

The original contributions presented in the study are publicly available. This data can be found here: https://www.ncbi.nlm.nih.gov/bioproject/PRJNA877374.

## Ethics statement

The animal study was reviewed and approved by Feed Research Institute, Chinese Academy of Agricultural Sciences Experimental Animal Ethics Committee.

## Author contributions

YW: funding acquisition and methodology. JW: writing–original draft and data curation. MJ: formal analysis and visualization. JL and YY: investigation. SH: conceptualization. YC: writing-reviewing and editing. TY: project administration. XG: supervision. All authors have read and agreed to the published version of the manuscript.

## Funding

This study was supported by the National Key Research and Development Program of China (Grant No. 2021YFD1300300), the Beijing Innovation Consortium of Poultry Industry Research System of China (BAIC04-2022), and the Agricultural Science and Technology Innovation Program of CAAS, China (CAAS-ASTIP-2022-FRI-08).

## Conflict of interest

The authors declare that the research was conducted in the absence of any commercial or financial relationships that could be construed as a potential conflict of interest.

## Publisher's note

All claims expressed in this article are solely those of the authors and do not necessarily represent those of their affiliated organizations, or those of the publisher, the editors and the reviewers. Any product that may be evaluated in this article, or claim that may be made by its manufacturer, is not guaranteed or endorsed by the publisher.
