# Supplementary material for: Clostridium autoethanogenum protein inclusion in the diet for broiler: Enhancement of growth performance, lipid metabolism, and gut microbiota
Source: Front Vet Sci. 2022 Nov 24;9:1028792. doi: 10.3389/fvets.2022.1028792 (PMC9731230; doi:10.3389/fvets.2022.1028792)
Supplement: Supplementary file 1 [file Data_Sheet_1.docx]

Supplementary Material

# Supplementary Figures and Tables

## Supplementary Tables 1

| Item | Starter period(1 to 21 d of age) | | | | | | | Finisher period(22 to 42 d of age) | | | | | |  |
| --- | --- | --- | --- | --- | --- | --- | --- | --- | --- | --- | --- | --- | --- | --- |
|  | T0 | T1 | T2 | T3 | T4 | T5 | T0 | | T1 | T2 | T3 | T4 | T5 | |
| Met | 0.22 | 0.23 | 0.24 | 0.25 | 0.26 | 0.27 | 0.20 | | 0.21 | 0.22 | 0.23 | 0.24 | 0.24 | |
| Cys | 0.23 | 0.22 | 0.22 | 0.21 | 0.20 | 0.19 | 0.21 | | 0.20 | 0.19 | 0.18 | 0.18 | 0.17 | |
| Lys | 0.98 | 1.01 | 1.04 | 1.06 | 1.09 | 1.11 | 0.89 | | 0.91 | 0.94 | 0.96 | 0.98 | 1.01 | |
| Thr | 0.63 | 0.63 | 0.63 | 0.63 | 0.63 | 0.63 | 0.57 | | 0.57 | 0.57 | 0.57 | 0.57 | 0.57 | |
| Trp | 0.22 | 0.21 | 0.20 | 0.19 | 0.19 | 0.18 | 0.20 | | 0.19 | 0.18 | 0.17 | 0.16 | 0.16 | |
| Arg | 1.12 | 1.09 | 1.06 | 1.02 | 0.98 | 0.94 | 1.03 | | 0.98 | 0.94 | 0.90 | 0.86 | 0.83 | |
| Ile | 0.71 | 0.73 | 0.73 | 0.74 | 0.75 | 0.76 | 0.65 | | 0.66 | 0.66 | 0.67 | 0.67 | 0.68 | |
| Leu | 1.18 | 1.17 | 1.16 | 1.15 | 1.14 | 1.13 | 1.07 | | 1.05 | 1.04 | 1.03 | 1.02 | 1.00 | |
| Val | 0.74 | 0.75 | 0.76 | 0.77 | 0.78 | 0.78 | 0.68 | | 0.68 | 0.68 | 0.69 | 0.70 | 0.70 | |
| His | 0.42 | 0.42 | 0.41 | 0.40 | 0.39 | 0.38 | 0.39 | | 0.38 | 0.36 | 0.35 | 0.34 | 0.33 | |
| Phe | 0.80 | 0.78 | 0.77 | 0.75 | 0.73 | 0.71 | 0.73 | | 0.70 | 0.68 | 0.67 | 0.65 | 0.63 | |
| Gly | 0.68 | 0.68 | 0.67 | 0.67 | 0.66 | 0.66 | 0.62 | | 0.61 | 0.60 | 0.60 | 0.59 | 0.59 | |
| Ser | 0.82 | 0.80 | 0.78 | 0.76 | 0.74 | 0.72 | 0.75 | | 0.72 | 0.70 | 0.68 | 0.66 | 0.64 | |
| Pro | 0.78 | 0.76 | 0.73 | 0.71 | 0.68 | 0.66 | 0.71 | | 0.68 | 0.65 | 0.63 | 0.60 | 0.58 | |
| Ala | 0.67 | 0.68 | 0.68 | 0.69 | 0.69 | 0.69 | 0.61 | | 0.61 | 0.61 | 0.62 | 0.62 | 0.62 | |
| Asp | 1.83 | 1.82 | 1.80 | 1.78 | 1.76 | 1.74 | 1.67 | | 1.64 | 1.61 | 1.59 | 1.57 | 1.54 | |
| Glu | 2.89 | 2.82 | 2.74 | 2.65 | 2.57 | 2.48 | 2.64 | | 2.53 | 2.44 | 2.35 | 2.26 | 2.17 | |
| Tyr | 0.00 | 0.03 | 0.06 | 0.09 | 0.13 | 0.16 | 0.00 | | 0.03 | 0.06 | 0.09 | 0.13 | 0.16 | |

**Supplementary Table 1.** Amino acid composition of experimental diets (Measured value) (%)

## Supplementary Tables 2

|  | Starter period(1 to 21 d of age) | | | | | | | Finisher period(22 to 42 d of age) | | | | | |  |
| --- | --- | --- | --- | --- | --- | --- | --- | --- | --- | --- | --- | --- | --- | --- |
| Ingredients, g/kg | T0 | T1 | T2 | T3 | T4 | T5 | | T0 | T1 | T2 | T3 | T4 | T5 |  |
| Corn | 540.4 | 551.2 | 554.5 | 555.1 | 556.1 | 556.1 | | 635.4 | 635.0 | 634.9 | 637.8 | 640.0 | 642.0 |  |
| Wheat Bran | 50.00 | 50.00 | 60.00 | 72.00 | 83.70 | 96.90 | | 0.00 | 15.00 | 29.00 | 39.00 | 49.00 | 60.00 |  |
| Soybean meal | 345.4 | 325.4 | 303.8 | 281.8 | 260.0 | 238.0 | | 315.4 | 291.3 | 268.3 | 246.0 | 224.0 | 201.4 |  |
| Ca(H_2_PO_4_)_2_ | 18.7 | 18.7 | 18.7 | 18.7 | 18.7 | 18.7 | | 13.0 | 13.0 | 13.0 | 13.0 | 13.0 | 13.0 |  |
| Lysine | 3.00 | 2.50 | 2.00 | 1.40 | 0.70 | 0.00 | | 0.50 | 0.40 | 0.00 | 0.00 | 0.00 | 0.00 |  |
| NaCl | 3.00 | 3.00 | 3.00 | 3.00 | 3.00 | 3.00 | | 3.50 | 3.50 | 3.50 | 3.50 | 3.50 | 3.50 |  |
| Methionin | 2.70 | 2.40 | 2.20 | 1.80 | 1.60 | 1.40 | | 0.70 | 0.50 | 0.30 | 0.00 | 0.00 | 0.00 |  |
| CAP | 0.00 | 10.00 | 20.00 | 30.00 | 40.00 | 50.00 | | 0.00 | 10.00 | 20.00 | 30.00 | 40.00 | 50.00 |  |
| Limestone | 13.60 | 13.60 | 13.60 | 14.00 | 14.00 | 14.00 | | 13.30 | 13.30 | 13.30 | 13.30 | 13.30 | 13.30 |  |
| Choline chloride | 1.00 | 1.00 | 1.00 | 1.00 | 1.00 | 1.00 | | 1.00 | 1.00 | 1.00 | 1.00 | 1.00 | 1.00 |  |
| Mineral premix^1^ | 2.00 | 2.00 | 2.00 | 2.00 | 2.00 | 2.00 | | 2.00 | 2.00 | 2.00 | 2.00 | 2.00 | 2.00 |  |
| Vitamin premix^2^ | 0.20 | 0.20 | 0.20 | 0.20 | 0.20 | 0.20 | | 0.20 | 0.20 | 0.20 | 0.20 | 0.20 | 0.20 |  |
| Soybean oil | 20.0 | 20.0 | 19.0 | 19.0 | 19.0 | 18.7 | | 15.0 | 14.8 | 14.5 | 14.2 | 14.0 | 13.6 |  |
| Total | 1000 | 1000 | 1000 | 1000 | 1000 | 1000 | | 1000 | 1000 | 1000 | 1000 | 1000 | 1000 |  |
| ME(MJ/kg)^3^ | 2.81 | 2.84 | 2.84 | 2.84 | 2.84 | 2.84 | | 2.93 | 2.92 | 2.92 | 2.92 | 2.93 | 2.93 |  |
| Analyzed nutrients compositions (g/kg, in dry matter basis) | | | | | | |  | | | | | | | |
| Moisture | 9.46 | 10.83 | 10.53 | 10.31 | 9.92 | 10.0 | | 9.55 | 9.46 | 9.85 | 10.10 | 10.05 | 9.96 |  |
| Crude ash | 62.2 | 66.4 | 67.6 | 68.4 | 67.7 | 65.9 | | 67.5 | 68.1 | 67.4 | 68.2 | 68.8 | 67.5 |  |
| Crude protein | 222.6 | 231.9 | 230.1 | 225.4 | 224.1 | 236.5 | | 209.7 | 209.3 | 210.2 | 210.9 | 211.2 | 211.0 |  |
| Ether Extract | 42.1 | 46.0 | 45.9 | 45.0 | 43.4 | 43.3 | | 4.29 | 4.30 | 4.30 | 4.30 | 4.30 | 4.29 |  |
| Gross energy (MJ/kg) | 20.74 | 20.24 | 20.41 | 20.30 | 20.15 | 20.24 | | 22.48 | 22.57 | 22.35 | 22.61 | 22.44 | 22.54 |  |

**Supplementary Table 2.** Composition and nutrient levels of basal diets (air-dry basis, g/kg).

^1^The mineral premix provided the following per kg of the diet for 1 to 21 days of age: Fe (as ferrous sulfate) 100 mg, Cu (as copper sulfate) 8.0 mg, Zn (as zinc sulfate) 100 mg, Mn (as manganese sulfate) 120 mg, I (as potassium iodide) 0.7 mg, Se (as sodium selenite) 0.3 mg. The mineral premix provided the following per kg of the diet for 22 to 42 days of age: Fe (as ferrous sulfate) 80 mg, Cu (as copper sulfate) 8.0 mg, Zn (as zinc sulfate) 80 mg, Mn (as manganese sulfate) 100 mg, I (as potassium iodide) 0.7 mg, Se (as sodium selenite) 0.3 mg.

^2^The vitamin premix provided the following per kg of the diet for 1 to 21 days of age: VA 10 000 IU, VD3 1000 IU, VE 20 IU, VK3 0.5 mg, VB1 2.0 mg, VB2 8.0 mg, pantothenic acid 10.0 mg, niacin 35.0 mg, VB6 3.5 mg, biotin 0.05 mg, folic acid 0.55 mg, VB12 0.01 mg. The vitamin premix provided the following per kg of the diet for 22 to 42 days of age: VA 8000 IU，VD3 750 IU, VE 15 IU, VK3 0.5 mg, VB1 2.0 mg, VB2 5.0 mg antothenic acid 10.0 mg, niacin 30.0 mg, VB6 3.5 mg, biotin 0.05 mg, folic acid 0.55 mg, VB12 0.01 mg.

^3^ME was calculated values.

CAP: Clostridium autoethanogenum protein.

## Supplementary Tables 3

| Item | Kit |
| --- | --- |
| TG | A110-2-1 |
| TC | A111-2-1 |
| TP | A045-2-2 |
| ALB | A208-1-1 |
| UA | C012-1-1 |
| SOD | A001-1-2 |
| GSH-Px | A005-1-2 |
| CAT | A007-1-1 |
| MDA | A003-1-2 |

**Supplementary Table 3.** Numbers of the kit purchased from Nanjing Jiancheng Bioengineering Institute (Nanjing, Jiangsu, China).

TG: Triglyceride; TC: total cholesterol; TP: total protein; ALB: albumin; UA: uric acid activities; SOD: enzymatic activities of Superoxide Dismutase; GSH-Px: Glutathione Peroxidase; CAT: Catalase; MDA: Malondialdehyde.
